# The Function of NM23-H1/NME1 and Its Homologs in Major Processes Linked to Metastasis

**DOI:** 10.1007/s12253-020-00797-0

**Published:** 2020-01-28

**Authors:** Barbara Mátyási, Zsolt Farkas, László Kopper, Anna Sebestyén, Mathieu Boissan, Anil Mehta, Krisztina Takács-Vellai

**Affiliations:** 1 Department of Biological Anthropology, Eötvös Loránd University, Pázmány Péter stny. 1/C, H-1117 Budapest, Hungary; 2 Department of Pathology and Experimental Cancer Research, Semmelweis University, 1st Budapest, Hungary; 3 Sorbonne Université, INSERM, Centre de Recherche Saint-Antoine, CRSA, F-75012 Paris, France; 4 Service de Biochimie et Hormonologie, AP- HP, Hôpital Tenon, Paris, France; 5 Division of Medical Sciences, Centre for CVS and Lung Biology, Ninewells Hospital Medical School, DD19SY Dundee, UK

**Keywords:** NDPK, Metastasis inhibitor, NM23, Phagocytosis, Apoptosis, Cell migration, Dynamin, Phosphohistidine, Cftr

## Abstract

Metastasis suppressor genes (MSGs) inhibit different biological processes during metastatic progression without globally influencing development of the primary tumor. The first MSG, NM23 (non-metastatic clone 23, isoform H1) or now called NME1 (stands for non-metastatic) was identified some decades ago. Since then, ten human NM23 paralogs forming two groups have been discovered. Group I NM23 genes encode enzymes with evolutionarily highly conserved nucleoside diphosphate kinase (NDPK) activity. In this review we summarize how results from NDPKs in model organisms converged on human NM23 studies. Next, we examine the role of NM23-H1 and its homologs within the metastatic cascade, e.g. cell migration and invasion, proliferation and apoptosis. NM23-H1 homologs are well known inhibitors of cell migration. *Drosophila* studies revealed that AWD, the fly counterpart of NM23-H1 is a negative regulator of cell motility by modulating endocytosis of chemotactic receptors on the surface of migrating cells in cooperation with Shibire/Dynamin; this mechanism has been recently confirmed by human studies. NM23-H1 inhibits proliferation of tumor cells by phosphorylating the MAPK scaffold, kinase suppressor of Ras (KSR), resulting in suppression of MAPK signalling. This mechanism was also observed with the *C. elegans* homolog, NDK-1, albeit with an inverse effect on MAPK activation. Both NM23-H1 and NDK-1 promote apoptotic cell death. In addition, NDK-1, NM23-H1 and their mouse counterpart NM23-M1 were shown to promote phagocytosis in an evolutionarily conserved manner. In summary, inhibition of cell migration and proliferation, alongside actions in apoptosis and phagocytosis are all mechanisms through which NM23-H1 acts against metastatic progression.

## Introduction: NM23-H1, the First Metastasis Suppressor

In the nineteen sixties, oxidative phosphorylation and histidine phosphorylation were competing theories in cellular energy synthesis. The latter process involves the lability of the phosphorylated nitrogens on histidine which creates a high energy phospho-intermediate that drives cellular thermodynamics. This review centres on a family of NM23/NDPK enzymes (nucleoside diphosphate kinases, see below) that synthesise such high energy phospho-histidines using trinucleotides such as ATP as donors. They are found within multiple cellular pathways and first considered as housekeeper enzymes. The gene encoding the first NM23/NDPK member was identified as a metastasis suppressor gene (MSG) inhibiting one or more steps of the mechanism whereby cancer cells gradually acquire independence from the primary tumor. These ordered sequential steps are called the metastatic cascade and the underlying genes differ from mutations globally affecting the initial growth of the primary tumor. The number of human metastasis suppressor genes is now numbered at about 30 [1, 2] but the first to be discovered was *NM23-H1* (non-metastatic clone 23, isoform H1) now renamed *NME1* (non-metastatic). *NM23-H1* was identified in 1988 by comparing a non-metastatic mouse melanoma cell line against its highly metastatic counterpart [3]. Specifically, the mouse isoform NM23-M1 was downregulated in the metastatic variant [3] and later experiments on various NM23 homologs showed reduced expression in metastatic cancer cells compared to their non-metastatic counterparts. This is a common feature of MSGs [1, 2]. The exact mechanism whereby NM23-H1 expression is lost in invasive tumors still remains to be elucidated. However, several mechanisms were proposed in this context, such as direct cleavage by lysosomal cathepsins [4], downregulation of NM23 expression through chromatin remodelling [5] or methylation of CpG islands on its promoter [6], ubiquitination leading to protein degradation [7] or silencing by miRNA [8].

Further complexity then arose because the human genome not only encodes ten NM23 (or NME) homologs, - divided into two groups based on sequence homology and enzymatic activity -, but many gene products share overlapping functions. The highly homologous group I isoforms (NM23-H1-H4 or NME1-4) all possess NDPK activity, whereas group II members (NM23-H5-H9 or NME5-9 and RP2, retinitis pigmentosa 2) are not only more divergent in sequence but also manifest little or no NDPK activity [9, 10]. It is now accepted that in melanomas and in epithelial tumors such as breast, liver, colon, and cervical carcinomas, NM23-H1 expression shows an inverse correlation with metastatic potential [11–20]. However, in hematological malignancies, ovarian and prostate cancer for example, the converse is observed, where an upregulated NM23-H1 level correlates with poor prognosis [21–23]. Studies in neuroblastoma also reported a positive correlation between NM23-H1 expression and tumor progression [24, 25]. Moreover, in aggressive cases a S120G missense mutation was identified, which seems to be specific for this tumor type [24, 26]. It is important to note that *NM23-H1* as a representing MSG is very rarely mutated in different tumor types, unlike tumor suppressor genes.

Besides the S120G mutation in neuroblastoma only loss-of-heterozygosity (LOH) was observed in colorectal carcinoma cases [27]. Rather the loss of NM23-H1 is typical in invasive tumors where its expression seems to be downregulated either at the transcriptional, translational or the posttranslational level by mechanisms suggested above [4–8]. A decrease in NM23-H1 expression in clinical specimens was also observed during the invasion process: at the invasive front of hepatocellular and colon carcinoma NM23-H1 staining was strongly reduced, whereas it remained intense in the central body of the primary tumor. These data argue for a dual regulation of NM23-H1 during tumor development and progression: overexpression of NM23-H1 can be detected in the primary tumor compared to the adjacent non tumoral tissue during early steps of tumorigenesis, and later a downregulation of NM23-H1 expression occurs during metastatic progression [28].

Importantly, NM23 members are pleiotropic in a number of ways relating to the controlling steps of the metastatic cascade. This includes cell migration [29], growth and differentiation [20, 30, 31], signal transduction, transcriptional regulation [32, 33], and apoptosis [34]. In addition, other molecular activities have been assigned to NM23/NDPKs, such as histidine-dependent protein kinase (histidine phosphotransferase) activity [35–38], unusual nuclease activity [39, 40], and lipid bilayer-binding [41]. Taken together, multiple functions have been assigned to the NM23/NDPK protein family and many interaction partners were identified by different *in vitro* methods [29].Thus *NM23* gene family display pleiotropic functions and their effect on metastatic progression might be contradictory in many cancers. These contradictory data might be due to the presence of highly homologous NM23 isoforms in cells, especially NM23-H1 and NM23-H2, which are most often not discriminated by antibodies and probes [42]. Conflicting data can also be explained by different cellular context, as NDPKs were proposed to act in opposing ways depending on the cellular environment where cells attached to a surface show behaviour that differs from cells in a growth medium [43]. In addition, it is unclear how loss of function of NDPK will manifest when they bound to partner proteins such as CFTR, whose mutation causes cystic fibrosis (CF). For example, in CF the works of R. Muimo in Sheffield, UK and others show that both NM23-H1 and NM23-H2 are present in a much larger complex of proteins bound to different domains of the CF gene product CFTR. These NM23 proteins are dysfunctional when CFTR is mutated. It is also observed that a six fold rise in cancer of the young exists in this disease.

In the latter case, CFTR acts as a signalling hub by approximating many partners, akin to the role of NDPK in Ras signalling complexes [44]. Emerging data suggest that other signalling pathways implicated in cancer progression, for example PI3K signalling may also have opposing outcomes in a context-dependent manner [45]. Therefore, experiments carried out in developmental models *in vivo* have been required to unravel the biological functions of NM23/NDPK family members (reviewed in [46]).

In this review we focus on two main issues: 1. we provide an overview on results obtained from model organisms that help to illuminate human NM23 data. 2. We emphasize three processes making up part of the metastatic cascade, e.g. cell migration and invasion, proliferation and apoptosis.

## Studies on Model Organisms Contributed to Reveal the Biological Functions of NM23/NDPKs

Model organisms often serve as proxies for understanding basic questions related to human diseases [47]. As NDPKs are highly conserved from yeast to human [10, 48], and almost identical around the catalytic site where the phosphorylated histidine resides, we and others were able to leverage the fewer number of isoforms found in model organisms. This paucity of *NM23* genes in flies, worms and amoebae has significantly contributed to the understanding of the biology in relation to human data. In the fruit fly, a mutation named Killer of prune (K-pn) causes no phenotype by itself but causes lethality in individuals homozygous for the nonlethal mutation prune (pn) which causes a prune (not red) color of the eye [49]. Thirty years later, the laboratory of Allen Shearn presented evidence that Killer of prune is a mutation in the *abnormal wing discs (awd)* gene identified through studies on imaginal disc development [50, 51]. Imaginal discs are sac-like epithelial structures that fail to form after *awd* deletion when *Drosophila* larvae try to develop into adult structures such as legs, antennae, wings, etc.

One year later, the *Drosophila* AWD was shown to be the homolog of NM23 (78% amino acid identity) [52]. Independently, an NDPK was cloned from the social amoeba *Dictyostelium discoideum*, and shown to be highly homologous to AWD and NM23 giving a function to these proteins [53, 54]. Parallel experiments monitored the enzymatic ‘activities’ of NM23 and AWD proteins finding that *awd* loss-of-function mutant larvae showed a strongly decreased NDPK activity because AWD provides ~ 98% of NDPK activity in *Drosophila* embryos [53]. The involvement of the NDPK activity in the *awd* phenotype was demonstrated *in vivo* in the fruit fly. This function is linked to a high energy N-phosphate linkage on histidine119 (histidine118 residue in human NM23) in the catalytic site [55]. Loss-of-function *awd* mutants cannot be complemented by the transgene carrying the H119A allele – which encodes a kinase dead enzyme -, only the wild-type *awd* cDNA-containing construct could rescue *awd*’s null mutation [55]. Additionally, the larval lethality phenotype of *awd* mutants was completely rescued by human *NM23-H2*, the most ancient NDPK, but not by its chromosomally adjacent human *NM23-H1* that arose by later gene duplication.

In the mouse, 8 NDPK paralogs have been identified, which can be divided into two distinct groups similar to human: NM23-M1-M4 isoforms are group I NDPKs, group II consists of M5-M8 isoforms [56]. NM23-M1 and NM23-M2 show 94% and 98% sequence identity with their human homologs, respectively.

To gain insight into NM23 proteins’ functions in a relatively close and highly relevant model organism to human, constitutive knockout mice have been generated for *NM23-M1* [57], *NM23-M2* [58], a double knockout of *NM23-M1* and *NM23-M2* [59], and from the group II *NM23-M5* and *NM23-M7* [60]. Single mutant mice for *NM23-M1* or *NM23-M2* are viable and beside a few developmental defects able to develop into adulthood. *NM23-M1* knockout mice develop a mild hypotrophy [57] and females display defective mammary gland growth with poor mammary duct maturation of the nipple [61]; as a result these females cannot feed their pups. The lack of *NM23-M2* affects the function of the immune system. In *NM23-M2* knockout mice T and B cell development is normal, but the T helper (Th) 1 and 2 cell types show insufficient cytokine production. In addition, in these CD4^+^ T cells the presence of the functional NM23-M2 is crucial for the K^+^ channel KCa3.1 activation, which occurs by phosphorylation through the histidine kinase activity of NM23-M2 [62]. *NM23-M1/NM23-M2* double knockout mice die perinatally [59] and suffer in hematological defects such as anemia with damaged maturation of erythrocytes, suggesting that NM23-M1 and NM23-M2 functions are indispensable for erythroid lineage development. It is likely that in double mutant mice, the level of triphospho-nucleoside precursors is insufficient for DNA and RNA synthesis. Genes from group II were characterized as components of axonemal structures of cilia and flagella [9]. Constitutive *NM23-M5* and *NM23-M7* knockout mice have already been generated [60, 63, 64], and showed situs inversus and hydrocephalus, respectively, phenotypes due to impaired motility of cilia.

Conditional knockout mice do not exist yet for NDPKs, but both conditional knockouts and specific tissue targeted deletions of murine NDPKs would significantly contribute to understand NDPKs’ function in the future.

## The Role of NM23-H1 in Processes Driving Metastatic Progression

### Cell Migration and Invasion

Primary tumor cells have to gain many capabilities in order to execute the metastatic program. One of the first characteristics needed is to acquire motility and invasion. Many biological functions are associated with the NM23/NDPK protein family, although not all of them are confirmed so far in *in vivo* systems. However, the ability of NM23-H1 to suppress cell migration and invasiveness is an accepted property [65].

Three types of evidence have been provided thus far to demonstrate the anti-metastatic activity of NM23-H1 or its mouse homolog NM23-M1. 1. Murine experiments by Boissan et al [66] proved the anti-metastatic effect of NM23-M1 as follows: crossing of transgenic mice prone to hepatocellular carcinoma (ASV mice expressing the SV 40 large T antigen in the liver leading to hepatocellular carcinoma), into an *NM23-M1* knockout strain, resulted in double transgenic mice showing a higher incidence of lung metastases compared to the original single mutants. 2. Overexpression of *NM23-H1/M1* in human or mouse invasive cell lines, where their endogenous expression in the parental line is low, resulted in reduction of the metastatic potential. For example, reduced migratory potential and decrease in invasiveness were observed in breast, colon, oral and hepatocellular carcinoma and different melanoma cell lines, when transfected with *NM23* transgenes [67–70]. 3. In a related manner, in response to silencing of *NM23-H1* in non-invasive cancer cell lines derived from hepatocellular carcinoma and colon cancer (where the endogenous NM23-H1 level is high), an invasive/metastatic phenotype was observed [28]. Intercellular adhesion was lost, cell motility and extracellular matrix invasion through type I collagen and Matrigel were increased, accompanied by upregulation of MT1-MMP (membrane-associated matrix metalloproteinase 1) expression, increased Rac1 signalling and MAPK (mitogen-activated protein kinase)/SAPK (stress-activated protein kinases) including ERK and JNK, and PI3K/Akt pathway activation [28]. Silencing of *NM23-H1* was associated with formation of invadopodia, defined as actin-driven membrane protrusions endowed with matrix proteolytic activity due to MT1-MMP, the key invadopodial metalloproteinase. In agreement with these data, invasion was inhibited by expression of a catalytic domain deletion mutant of MT1-MMP in silenced cells. Conversely, overexpression of the proteolytically active form of MT1-MMP promoted invasion in control cells and led to further increased invasion of silenced cells. Several mechanisms could explain how NM23-H1 inhibits cell migration. These data centre on the negative regulation of Rho-Rac signalling by NM23-H1. Rac1 is a pleiotropic regulator of cell motility, and the above experiments suggest that Rac1 activity is inhibited by NM23-H1. In line with these data, Tiam1, a Rac1-specific nucleotide exchange factor was shown to be negatively regulated by NM23-H1, resulting in attenuated Rac1 activation [71]. NM23-H1 was also shown to bind to Dbl-1, a specific exchange factor of a Rho-type GTPase, Cdc42. Binding of NM23-H1 to Dbl-1 inactivated Cdc42 and hence inhibited cell migration [72]. Interestingly, reduced transcription of *EDG2* encoding a lysophosphatidic acid receptor can also lead to decreased cell motility. Downregulation of *EDG2* expression can be obtained by increasing expression of *NM23-H1* [73]. Recently, an inverse correlation between EDG2 and NM23 expression was also observed during myeloid differentiation [74].

Several other important mechanisms were proposed by which NM23-H1 exerts its effect on cell migration. First, NM23-H1 was shown to bind to gelsolin and inactivate its actin-severing capacity in order to suppress tumor cell motility and metastasis [75]. Second, the puzzling *awd/prune* interaction known from the fruit fly (see above) was further investigated in breast cancer and it was found that NM23-H1 directly interacts with the phosphodiesterase h-Prune [76] resulting in increased cell motility due to an inhibitory sequestrative interaction with NM23-H1. The mechanism causing increased motility of breast cancer cells was also explored by the formation of the NM23-H1/hPrune complex, which is induced through phosphorylation of NM23-H1 by casein kinase I in specific Ser residues [77]. In colon cancer cells NM23-H1 was found to inhibit cell migration via phosphorylation of the myosin light chain (MLC) [78]. TGF-β signalling is a major inducer of epithelial-mesenchymal transition. TGF-β treatment of lung cancer cells resulted in increased invasive and migratory potential, which was further enhanced by *NM23-H1* knockdown, meaning that NM23-H1 emerges as a new upstream factor acting against epithelial-mesenchymal transition [79]. Thus, it appears that multiple mechanisms induce a strong invasive and motile phenotype in response to *NM23-H1* silencing, in agreement with the multifunctional properties of this metastasis-suppressor protein. This points also to a role of NM23-H1 as a major upstream regulator of the metastatic signaling cascade.

Complementary *Drosophila* studies in the Hsu lab revealed a new, distinct mechanism whereby AWD, the fly counterpart of mammalian NM23-H1 regulates cell migration.

AWD was the first NDPK whose role was examined in *in vivo* cell migration models: AWD’s function was investigated in two well characterised migrating cell types during *Drosophila* development: in migration of tracheal cells and border cells. Fly tracheogenesis is a model of tubular morphogenesis [80, 81]. The process starts during early embryogenesis by emergence of tracheal placodes, an invagination of specialized ectodermal cells, from which tracheal branches later develop. Cell migration and fusion events will give rise to the tracheal network as tracheal branches elongate, migrate and fuse to form tracheal tubules (branching morphogenesis). FGF (fibroblast growth factor) is the key diffusive chemotactic signal, which drives tracheal cell migration: migrating tracheal cells express fibroblast growth factor receptor (FGFR) on their surface and follow FGF, the signal released by the surrounding tissues [80]. AWD was found to negatively regulate tracheal migration with low AWD dosage correlating with ectopic migration of tracheal cells. Hsu and colleagues proposed that AWD acts as a negative regulator of cell motility through modulating FGFR levels on tracheal cell surfaces by regulating endocytosis of FGFR [82].

A similar approach can use border cell migration during *Drosophila* oogenesis [83]. The egg chamber, a structure in the fly ovary producing a given egg, is composed of the oocyte and surrounding nurse cells (the so-called germ cell complex), itself surrounded by a somatic epithelium of follicle cells. Border cells are a special group of 6–10 follicular cells which delaminate from the somatic epithelium and migrate to the anterior pole of the oocyte to form the micropyle (entry point of sperms) [83]. Although AWD is expressed in the follicular epithelium, its expression is downregulated in border cells, which permits their migration [84]. Conversely, overexpression of AWD results in stalled migration of border cells, suggesting that AWD dosage inhibits the motility of these cells as well [84]. Ligands of PDGF (platelet derived growth factor) receptor and JAK/STAT pathways are known to provide chemotactic signals for border cells [85–87]. Once again, AWD influences the level of VEGF (vascular epithelial growth factor)/PDGF receptors and JAK/STAT homologs (called Pvr and Domeless, respectively) on the surface of border cells through regulating receptor endocytosis, as seen in tracheogenesis [84].

Importantly AWD partners Shibire, the homolog of human Dynamin in these functions. This idea sits well with independent data where AWD was identified as an exclusive binding partner of Shibire/Dynamin during studies on Dynamin’s function in synaptic vesicle recycling [88].

The data suggest that Dynamin (an atypical large GTPase) plays a key role in the initiating step of endocytosis: Dynamin is essential for generation of endocytic vesicles as it accumulates around the neck of the invaginated cell membrane by forming a polymerized helix [89]. GTP hydrolysis by the Dynamin polymer leads to neck constriction and subsequent vesicle fission. This model is supported by data from *Drosophila* researchers showing that AWD functions as an exclusive GTP supplier towards Shibire/Dynamin [88]. The mechanistic basis of the Dynamin/NDPK interaction was provided by studying NM23’ role in endocytosis of several receptors in human and monkey cell lines: NDPKs fuel Dynamin locally by GTP to make it possible to work at high thermodynamic efficiency [90]. More recently, this idea has been expanded by the Steeg lab that reports that NM23-H1 interacts with Dynamin in MDA-MB-231 breast carcinoma cells and the above mentioned NDPK contribute to suppression of tumor cell motility by promoting endocytosis of chemotactic receptors by facilitating Dynamin oligomerization and increasing its GTPase activity [91].

Together, *Drosophila* studies are consistent across model systems of migration revealing that the Dynamin/NDPK complex plays an essential role in suppression of cell motility by downregulating chemotactic receptor levels through receptor internalization on the surface of migrating cells, further confirmed in human studies.

### NM23/NDPK’s Role in Proliferation: NM23-H1 Influences the Outcome of the Ras/MAPK Cascade by Interacting with KSR and the Ratio of ERK/p38 Signal Activity

Receptor tyrosine kinase (RTK)/Ras/extracellular signal regulated kinase (ERK) signalling functions in many important biological processes, for example in cell proliferation, differentiation, cell migration and survival (reviewed in [92]). The RTK/Ras/ERK pathway contributes significantly to proliferation of tumor cellsas mutations in pathway components often lead to tumorigenesis [93]. Kinase suppressor of Ras proteins (KSRs) are scaffolds which promote the assembly of Raf/MEK/ERK complexes [94–96] and enable phosphorylation of MAPK cascade components. KSR1 can bind all the members of the MAPK pathway: it binds constitutively to MEK and approximates MEK upon Ras activation to the plasma membrane adjacent to Raf, thereby facilitating signal transduction. KSR scaffolds can themselves be regulated by phosphorylating critical Ser residues. For example p-Ser392 promotes binding of KSR to 14-3-3 proteins, which in turn results in cytoplasmic sequestration and inactivation of the scaffold [97]. Conversely, Ser392 dephosphorylation results in KSR activation, allowing its localization to the plasma membrane. The phosphatase PP2A, and two kinases, Cdc25C-associated kinase 1 (C-TAK1) and NM23-H1 were found to operate on Ser392 residue to regulate cellular localization and thus activation of KSR [97, 98]. Phosphorylation of Ser392 by NM23-H1 occurs through its histidine kinase activity [37]. The effect of NM23-H1 exerted on MAPK signalling was also examined in MDA-MB-435 breast carcinoma cells where *NM23-H1* overexpression leads to KSR phosphorylation and decreased activated MAPK (pERK) levels. Conversely, silencing of *NM23-H1* in HepG2 cells leads to hyperactivation of ERK [28]. These data imply that NM23-H1 negatively regulates the outcome of Ras/MAPK signalling.

The KSR/NDPK interaction was confirmed *in vivo* in the tractable genetic model *Caenorhabditis elegans* [99]. The function of the nematode group I NDPK homolog NDK-1 was shown to alter the size and composition of the hermaphrodite reproductive organ, the vulva [99]. Vulval development is a complex, highly cell-ordered set of events involving a cascade of interacting pathways that includes EGFR/Ras/MAPK pathway, together with Notch, Wnt and the Synthetic Multivulva (SynMuv)). Their coordinated actions are essential when vulval precursor cells (VPCs), a subset of epidermal blast cells, develop the competence to form the vulval tissue. The ligand EGF (epidermal growth factor), which initiates differentiation, is produced by a specific cell of the somatic gonad, the anchor cell (AC). EGF activates the EGFR/Ras/MAPK pathway in the three closest VPCs, P(5–7).p (reviewed in [100, 101]). These induced VPCs each divide three times and give rise to the 22 daughter cells that will form the adult vulval structure following cell migration and fusion events. Different phenotypic alterations can occur due to defects in EGFR/Ras/MAPK signalling: lack of Ras signal leads to the Vulvaless phenotype (absence of vulva), whereas increased Ras signalling causes a Multivulva phenotype (’excess’ vulva development, reviewed in [100]). *ndk-1* null mutants display a Protruding vulva (Pvl) phenotype, due to the eversion of the vulval tissue [102]. Epistasis analysis was used to position *ndk-1* in the process of vulval patterning and *ndk-1* was found to act downstream of *lin-45/Raf* but upstream of *mek-2/MEK* and *mpk-1/MAPK* in the EGFR/Ras/MAPK pathway [99]. In line with these data, a significant decrease was detected in activated phospho-MAPK levels in somatic tissues of *ndk-1* knockouts, accompanied by unchanged total MAPK levels. Moreover, activated MAPK was not detectable in the germline of mutants, suggesting that Ras/MAPK signalling is inhibited in the absence of NDK-1 [99].

In *C. elegans*, the scaffold proteins KSR-1/KSR-2 are generally indispensable for MAPK activation [103] and based on the above data NDK-1 also contributes to full activation of MAPK signalling. Hence, *ndk-1(-);ksr-1(-)* and *ndk-1(-)ksr-2(-)* double knockouts were generated. Both double mutants showed enhanced single mutant phenotypes, verifying the genetic interactions between *ndk-1* and *ksr* genes. NDK-1 also showed a physical interaction with worm KSR-2 and murine KSR-1 by *in vitro* pulldown experiments [99]. Thus, a previously proposed link [37, 104] was confirmed *in vivo* between an NDPK and KSR scaffolds [99] but the effect differs between species. In human studies, NM23-H1 attenuates Ras/MAPK signalling through phosphorylation of KSR [37, 104], in contrast, worm NDK-1 promotes the Ras/MAPK pathway as it is required for proper MAPK activation [99]. One hypothesis for future study suggests that this discrepancy might be due to a differential regulation of the KSR scaffold.

Activation of RTK/Ras/MAPK pathway as a main proliferation signal plays an important role at a late step of the metastatic cascade, when tumor cells leave the primary tumor, arrive to a new environment through the circulation and form a new tumor focus at the secondary site.

**Fig. 1:**
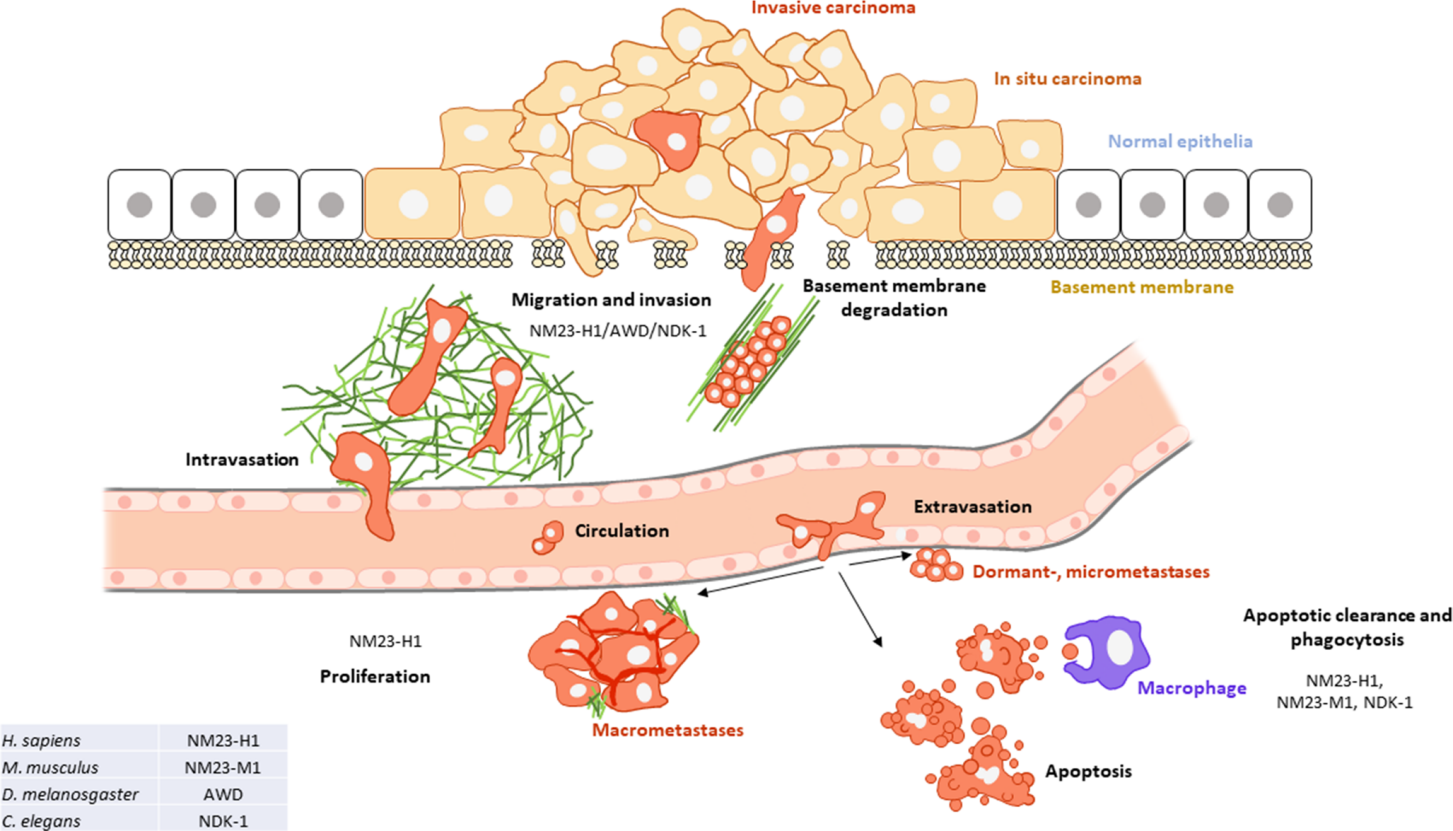
The role of NM23-H1, NM23-M1, AWD and NDK-1 in processes related to the metastatic cascade. Metastasis formation is a complex and multistep process, as a tumor cell acquires invasive characteristics and passes through the steps of the metastatic cascade. In case of carcinomas, tumor cells of epithelial origin detach from the primary tumor, gaining access to the surrounding stroma by breaching the basement membrane (invasion), then they enter the microvasculature of the lymphatic or blood system (intravasation). Next they survive in the circulation avoiding immune surveillance and translocate to distant tissues. Following extravasation tumor cells (micrometastases) have to survive in a new microenvironment of the distant tissue. After cancer cells have spread from the primary tumor to a secondary site, just a small number of them can conform to this new environment and form micro- and macrometastases. The majority of these tumor cells step into a dormant state or forced into apoptotic cell death by the signals from the new microenvironment. Successful metastatic colonization with formation of macrometastases often depends on avoidance of apoptotic signals and employing proliferation signals by the micrometastases. Subsequently secondary colonies will be eventually able to establish new blood supplies. If any of these steps fail to occur, metastases cannot develop. In this review we emphasize three major processes in which NM23-H1 and its homologs, AWD (D. melanogaster), NDK-1 (C. elegans) and NM23-M1 (M. musculus) have an important role. These processes are linked to different steps of the metastatic cascade. NM23-H1, AWD, and NDK-1 inhibit cell migration (NM23-H1 suppresses invasion as well). NM23-H1 was shown to inhibit proliferation. NM23-H1, NM23-M1 and NDK-1 promote phagocytosis, whereby apoptotic cell debris are eliminated by macrophages. Cell migration plays a role in an early step of the metastatic cascade, whereas proliferation and apoptosis are processes influencing a late step, metastatic colonization.

If disseminated tumor cells (also called micrometastases) are able to adapt to the new challenging microenvironment, can start to proliferate and form a secondary tumor focus. Successful metastatic colonization with formation of macrometastases often depends on employing proliferation signals and avoiding apoptotic signals by the micrometastatic cells [105, 106]. Micrometastases can develop basically into three different directions: 1. Micrometastases undergo cell death if they are unable to resist to apoptotic signals. 2. Metastatic dormancy frequently occurs when micrometastatic dormant cells are able to survive but do not proliferate. In this case apoptotic vs proliferative signals are balanced. Metastasis suppressor genes contribute to metastatic dormancy by participating in the above processes [107]. 3. Successful colonization to macrometastases happens if micrometastatic cells avoid apoptosis and manage to proliferate (Fig. 1). The ratio of ERK/p38 signal activity is known to contribute to successful development of macrometastases from disseminated tumor cells: high ERK/p38 activated ratio favours tumor growth, whereas high p38/ERK activated ratio induces tumor growth arrest (dormancy) *in vivo* [108]. The expression of *EDG2* or *LPA1* (lysophosphatidic acid receptor 1) gene shows an inverse correlation with that of *NM23-H1*. The effect of LPA1 inhibition (which is attached to *NM23-H1* overexpression) was examined in an experimental pulmonary metastasis mouse model, and it was found by Marshall et al. [109] that the LPA1 inhibitor Debio-0719 induced high p38/ERK activated ratio in the lung, indicative of metastatic dormancy at the secondary site (reviewed in [110]).

### The Role of NM23/NDPKs in Apoptosis, Apoptotic Engulfment and Phagocytosis

In recent decades of study on the sequential steps underpinning the metastatic cascade, it has been established that type I programmed cell death or apoptosis is a barrier that tumor cells have to overcome in order to survive and proliferate (reviewed in [111–113]). Based on this logic, NM23 acts against tumor progression in two different ways: 1. in several cell types it was shown to act in the dying cell to promote cell death; 2. it functions in any engulfing cell with phagocytic capacity. The purpose of the latter is to eliminate the debris of the apoptotic cell in order to prevent inflammation, given that inflammation is a factor that might favour tumor progression [111].

Both human and *C. elegans* studies suggest that NDPKs play a role in several cases in the apoptotic cell. A recent study in *C. elegans* showed that germ cell death in response to DNA damage is mediated by NDK-1 through activation of the MAPK cascade [114]. NDK-1 acts as an activator of the MAPK pathway in somatic tissues of the worm; and this function of NDK-1 was confirmed from a different aspect, during germ cell apoptosis [114]. DNA damage-induced apoptosis of *C. elegans* germ cells is mostly driven by the MAPK pathway [115, 116]. *mir-35* miRNA has an important function in this process, as absence of *mir-35* leads to elevated levels of activated (phosphorylated) MAPK. The 3’-UTR of *ndk-1* contains a single *mir-35* binding site, where *mir-35* is able to inhibit translation of *ndk-1*, thus MAPK pathway’s activation is retained. In response to irradiation in the *C. elegans* germline (applying genotoxic stress), elevated NDK-1::GFP levels were experienced in germ cells destined to die; especially, if the *mir-35* binding site was mutated in *ndk-1*’s 3’-UTR, the refractile morphology of apoptotic germ cells suddenly occurred. The authors conclude that *mir-35* acts as a buffer to maintain the threshold of apoptosis, by influencing MAPK activation through NDK-1 [114].

Earlier studies already linked NDPKs to another specialized type of cell death: human NM23-H1 acts in caspase-independent apoptosis as a granzyme A-activated DNAse (GAAD) [34, 117]. Similar to the earlier trends showed, those granzyme A-treated cells in which NM23-H1 is overexpressed, turned out to be more sensitive to granzyme A-mediated DNA damage and cytolysis.

Another interesting set of data linked NM23-H1 to apoptosis found by Boissan et al: HepG2 cells treated by siRNA specific for *NM23-H1* showed reduced sensitivity to chemotherapeutic agent-induced apoptosis [28].

In multicellular organisms dying cells are removed by neighbouring cells or specialized phagocytes, which engulf the apoptotic cells and destroy their debris. The internalized apoptotic cells are subsequently degraded inside phagosomes, which undergo a multistep process called phagosome maturation that involves the fusion of phagosomes and multiple kinds of intracellular organelles accompanied by the gradual acidification of the phagosomal lumen [118]. The presence of uncleared apoptotic cells has been associated with different diseases that involve inflammation, autoimmunity and cancer [119]. As a result of insufficient removal of apoptotic cells, chronic inflammation occurs, which might favour tumor growth and progression [120]. In context of apoptotic cell clearance (2. point above) and phagocytosis, we investigated NDPKs function in phagocytosis in *C. elegans*: in the absence of NDK-1 accumulation of apoptotic cell corpses was observed, both in embryos and in the gonad of mutant hermaphrodites [121]. NDK-1::GFP expression was detected in the gonadal sheath cells, which are specialized engulfing cells of dying germ cells. Importantly, a genetic interaction occurred between *ndk-1* and *dyn-1* (dynamin), which was later confirmed at the protein level with multiple IP experiments and *in situ* with DPLA (duolink proximity ligation assay) [122]. Furthermore, NDK-1 acts in the same time-window as DYN-1 on phagosomal surfaces, during engulfment and the early steps of phagosome maturation. This connection turned out to be conserved; DPLA confirmed the co-localisation of NM23-H1 and Dynamin-2 in human macrophages as well, and increased enrichment of the two proteins was shown at the phagocytic cups. Depleting *NM23-H1* in macrophages led to decreased phagocytic capacity. Similarly, murine BMDMs (bone marrow–derived macrophages), due to *NM23-M1* silencing engulfed significantly less apoptotic thymocytes [122].

Thus, NM23/NDPKs role in phagocytosis is evolutionary conserved. NM23-H1, NM23-M1 and NDK-1 are all factors promoting phagocytosis [122].

## Conclusion

In this review we examined the role of NM23-H1 and its homologs in three processes making part of the metastatic cascade, e.g. cell migration and invasion, proliferation and apoptosis.

Cell invasion and migration play an important early role in the metastatic cascade: capability of invasion and migration are among the first features primary tumor cells need to acquire in order to evolve into metastases. Several pro-invasive signalling pathways are inhibited by NM23-H1, including the Rac1 pathway, MAPK/SAPK, PI3K/Akt. NM23-H1 inhibits formation of invadopodia and thus proteolysis of extracellular matrices through modulation of MT1-MMP expression. NM23-H1 homologs are well known inhibitors of cell migration (Fig. 1). Human studies linked NM23-H1 to cell migration inhibition through modifying Rho-Rac and TGFβ signalling and actin remodeling. Two *in vivo* cell migration models served to examine the role of AWD, the fly counterpart of NM23-H1, in regulating cell motility in *Drosophila*, tracheogenesis and border cell migration. These studies revealed that AWD is a negative regulator of cell motility through modulating endocytosis of chemotactic receptors on the surface of migrating cells in cooperation with Shibire/Dynamin. Recent work by Patricia Steeg and colleagues shows that NM23-H1 facilitated Dynamin activity, thereby promoting endocytosis of chemotactic receptors and suppressing tumor cell motility.

In the above fly and human studies endocytosis of the following receptors were examined: FGFR (tracheal migration), VEGFR/PDGFR (border cell migration), EGFR (studies on human cell lines). We note that all these receptors play a role as deregulated factors involved and detected in many different cancer types. Thus, they represent therapeutic targets in personalized medicine [123].

NM23-H1 might function in a late step of the metastatic cascade as well by inducing tumor dormancy when apoptotic and proliferative signals are balanced. NM23-H1 inhibits tumor cell proliferation by phosphorylating the MAPK scaffold KSR resulting in suppression of MAPK signalling. Overexpression of *NM23-H1* was shown to promote metastatic dormancy in a pulmonary metastasis mouse model. Although the exact mechanism remains to elucidate, in this mouse model a high activated p38/ERK ratio was found meaning that proliferative ERK signalling was indeed suppressed in response to *NM23-H1* overexpression.

Both NM23-H1 and its *C. elegans* homolog NDK-1 were found to promote apoptotic cell death of several cell types, although this issue needs further investigation. In addition, NM23-H1, its mouse and worm homologs function in engulfing cells to eliminate debris of the apoptotic cells, as they promote phagocytosis in an evolutionary conserved manner. Apoptotic clearance is an important issue from cancer perspective, as the accumulation of apoptotic cell corpses can trigger inflammation, which creates a supporting environment for tumor progression [111, 124].

NM23-H1 and its homologs were shown to be involved in the above processes (Fig. 1 and Table 1). Inhibition of cell invasion/migration and proliferation, and helping apoptosis and phagocytosis are all mechanisms, whereby NM23-H1 acts against metastatic progression.

**Table 1:** NM23-H1 and its *M. musculus*, *D. melanogaster* and *C. elegans* counterparts participate in three main processes linked to the metastatic cascade

	Organism	Factor	Regulated pathways	Function and biological role	Citation
Migration and invasion	Human tumor (e.g. breast, colon, oral, hepatocellular carcinoma and melanoma) cell lines	NM23-H1	Rho - Rac1 signaling MAPK/SAPK and PI3K/Akt pathway downregulation Gelsolin hPrune EDG2 MLC TGFβ	Inhibition of cell motility and invasion	[67–70] [71–73] [28] [75] [76–77] [73] [78] [79]
	*D. melanogaster*	AWD	FGFR PDGFR/VEGFR	Tracheogenesis and border cell migration	[82] [84]
	*M. musculus*	NM23-M1		Increased incidence of lung metastases in *NM23-M1* KO mice prone to develop hepatocellular carcinoma	[66]
Proliferation	Human breast carcinoma cell line	NM23-H1	Suppression of Ras-MAPK signalling through KSR	Proliferation	[37] [104]
	*C. elegans*	NDK-1	Activation of Ras-MAPK signaling through KSR	Vulval development	[99]
Apoptotic engulfment and phagocytosis	Human macrophages (primary cell culture)	NM23-H1		Phagocytosis of zymosan and IgG-opsonized RBCs	[122]
	*M. musculus* bone marrow-derived macrophages	NM23-M1		Engulfment of apoptotic thymocytes	[122]
	*C. elegans*	NDK-1		Apoptotic clearance of embryonic and germ cells	[121] [122]

Three main processes (cell migration and invasion, proliferation, apoptotic engulfment/phagocytosis) linked to the metastatic cascade where NM23-H1 and its homologs in model organisms play an important role. Biological functions and the molecular background are also indicated if known. Related citations are listed. See details in the text
